# The Principles of Surgery

**Published:** 1845-04

**Authors:** 


*The Principles of Surgery.* By James Miller, F. R. S. E., F. R. C. S. E., Professor of Surgery in the University of Edinburg, Surgeon to the Royal Infirmary, &c. &c. 8vo. pp. 525. Lea & Blanchard, Philadelphia, 1845.

Unlike most American reprints of British works, the present contains no "notes and additions," but is a simple transcript, if we may so employ the term, of the work, as it was put forth by the author.

At a period when the minds of the less reflecting part of the profession seem to be deluded with the notion that *cutting* constitutes *Surgery,* it is refreshing to find a distinguished member of the craft stepping forward as the advocate of principles, the proper application of which may not only guide but supersede that terrible remedy. The careful study of the work before us, is well calculated to subserve these purposes. From the Preface we learn that it comprises the substance of the lectures delivered by Professor Miller on the subject, before the class of the renowned University of Edinburgh. In its preparation, "it has been his aim to combine, with soundness of doctrine, such simplicity of arrangement, and plainness of illustration, as seem best calculated to facilitate, while they direct, the labours of the student." The work is divided into four sections.

The first section is occupied with the consideration of "ELEMENTARY DISEASE," and embraces various chapters: 1. *Perverted action of the blood-vessels*—i. e.—Inflammation, Congestion. 2. *Perverted action of the Nerves—Irritation* (local and constitutional,) —the shock of Injury. 3. *Perverted actions of the absorbents*—Atrophy, Interstitial Absorption, Continuous Absorption. 4. *Suppuration*—Abscess, acute, chronic, and diffuse—scrofula. 5. *Ulceration*—Granulation, Cicatrisation, Ulcers. 6. *Mortification.*

Section 2. TREATS OF PERVERTED VASCULAR ACTION IN CERTAIN TISSUES. Chapter 7. *Of Perverted action in the Integuments.* 8. *Perverted action occurring in bone.* 9. *Diseases of the Joints.* 10. *Diseases of the Arteries.* 11. *Affections of the Veins.* 12. *Hæmorrhage.* 13. *Affections of the Lymphatics.* 14. *Affections of Nerves.*

Sect. 3. TREATS OF PERVERTED NUTRITION. Chapter 15. Of tumours—benign, malignant, encysted, of the integument, of mucous membrane, of nerve, of bone.

Sect. 4. TREATS OF INJURIES. Chapt. 16. Wounds—incised, contused and lacerated, punctured, poisoned, gunshot, subcutaneous,—tetanus. 17. *Burns and Scalds.* 18. *Effects of Cold.* 19. Fracture. 20. *Dislocation.* 21. *Sprain and rupture of muscle and tendon.* 22. *Bruise.*

It will be seen from this enumeration of subjects, that Professor Miller's work embraces abundant materials for a full developement of the principles of Surgery, and we feel no hesitation in expressing our opinion, that it presents the philosophy of the science more fully and clearly than any other work in the language with which we are acquainted.

